# Fast, Accurate, and Robust T2 Mapping of Articular Cartilage by Neural Networks

**DOI:** 10.3390/diagnostics12030688

**Published:** 2022-03-11

**Authors:** Gustav Müller-Franzes, Teresa Nolte, Malin Ciba, Justus Schock, Firas Khader, Andreas Prescher, Lena Marie Wilms, Christiane Kuhl, Sven Nebelung, Daniel Truhn

**Affiliations:** 1 Department of Diagnostic and Interventional Radiology, University Hospital Aachen, 52074 Aachen, Germany; gustav.mueller-franzes@rwth-aachen.de (G.M.-F.); tnolte@ukaachen.de (T.N.); malin.ciba@rwth-aachen.de (M.C.); firas.khader@rwth-aachen.de (F.K.); ckuhl@ukaachen.de (C.K.); snebelung@ukaachen.de (S.N.); dtruhn@ukaachen.de (D.T.); 2 Department of Diagnostic and Interventional Radiology, University Hospital Düsseldorf, 40225 Düsseldorf, Germany; justus.schock@med.uni-duesseldorf.de; 3 Institute of Molecular and Cellular Anatomy, RWTH Aachen University, 52062 Aachen, Germany; aprescher@ukaachen.de

**Keywords:** neural network, quantitative MRI, T2 relaxometry, mono-exponential least-squares fitting, patellofemoral cartilage, osteoarthritis

## Abstract

For T2 mapping, the underlying mono-exponential signal decay is traditionally quantified by non-linear Least-Squares Estimation (LSE) curve fitting, which is prone to outliers and computationally expensive. This study aimed to validate a fully connected neural network (NN) to estimate T2 relaxation times and to assess its performance versus LSE fitting methods. To this end, the NN was trained and tested in silico on a synthetic dataset of 75 million signal decays. Its quantification error was comparatively evaluated against three LSE methods, i.e., traditional methods without any modification, with an offset, and one with noise correction. Following in-situ acquisition of T2 maps in seven human cadaveric knee joint specimens at high and low signal-to-noise ratios, the NN and LSE methods were used to estimate the T2 relaxation times of the manually segmented patellofemoral cartilage. In-silico modeling at low signal-to-noise ratio indicated significantly lower quantification error for the NN (by medians of 6–33%) than for the LSE methods (*p* < 0.001). These results were confirmed by the in-situ measurements (medians of 10–35%). T2 quantification by the NN took only 4 s, which was faster than the LSE methods (28–43 s). In conclusion, NNs provide fast, accurate, and robust quantification of T2 relaxation times.

## 1. Introduction

Cartilage degeneration is the hallmark change of osteoarthritis, which is a widespread degenerative disorder that affects the entire joint with enormous individual and socio-economic disease burden [1]. MRI offers unparalleled soft-tissue contrast and spatial resolution, while being non-invasive and lacking ionizing radiation. Therefore, MRI is the clinical reference standard for suspected joint and/or cartilage pathologies [2]. Yet, early—and potentially reversible—degeneration is often missed, which may explain the variable sensitivities of 45–74% for clinical-standard morphologic MRI techniques in the detection of cartilage lesions [3,4]. Considering this limitation in reliably confirming (or ruling out) early cartilage degeneration, quantitative MRI techniques such as T2 mapping have been evaluated in a range of scientific and clinical contexts [5,6,7]. T2 mapping techniques are robust, validated, and associated with biologically meaningful tissue properties, even though changes in T2 are not related to a single tissue property but rather reflective of changes of the collagen content, collagen network organization and integrity, and water content [8]. Recent longitudinal data confirmed the prognostic value of T2 maps as an imaging biomarker of cartilage because elevated T2 relaxation times have been shown to indicate future development of morphologic cartilage lesions and osteoarthritis [9].

Traditionally, T2 maps are generated by determining the voxel-wise signal decay based on a series of spin-echo images acquired at different echo times. For each voxel, the T2 relaxation time is then calculated by fitting a mono-exponential decay equation to the measured signal intensities, which is commonly performed by Least-Squares Estimation (LSE) [10,11,12]. Different MRI sequences are available for T2 quantification. A multi-echo spin-echo (MESE) sequence, for example, albeit being prone to different confounding factors such as stimulated echoes, the slice profile, or deviating refocusing angles [13,14,15], is, overall, faster than acquiring a series of single spin-echo images and present on most clinical scanners. However, high-speed or high-resolution images that are acquired for clinical purposes are generally contaminated with noise, which decreases the signal-to-noise ratios (SNRs) substantially [16]. Fitting the mono-exponential decay equation to these low-SNR images may skew the resultant T2 relaxation times [17] and lead to their overestimation by up to 500% [12]. Therefore, accuracy of LSE fitting can be substantially impaired in low-SNR images. Furthermore, LSE fitting methods are slow, computationally expensive [11], and prone to outliers, which reduces their robustness [18,19].

In the past, several methods were proposed to minimize the associated estimation errors for T2 fitting. Koff et al. compared linear, weighted, and non-linear fitting algorithms and found significant differences of up to 4.5 ms in the retropatellar cartilage of 10 healthy participants, thereby highlighting the fact that T2 relaxation times are substantially affected by the underlying method of fitting [20]. As the mean difference in T2 relaxation times between normal and abnormal cartilage and, thus, between healthy individuals and patients with (mild) osteoarthritis may be as low as 1.9 ms [21], the diagnostic distinction of health and disease may be even more challenging during clinical image post-processing and decision-making if the method of T2 map reconstruction is prone to noise or otherwise not well standardized. Consequently, the small differences in T2 values between healthy and mildly diseased cartilage may lead to failed diagnostic distinctions due to the estimation gap between the fitting techniques.

With the advent of ever-increasing computational power, artificial neural networks (NNs) are increasingly applied in the context of medical image acquisition and post-processing [22]. NNs have been used for robust parameter fitting, and their validity has been demonstrated in the presence of low SNRs and outliers [18,23]. In the context of T2 mapping, NNs have been used for generating T2 maps from under-sampled k-space data [24,25], and for multi-exponential fitting of T2 relaxation times in the brain [11,26]. However, to the best of our knowledge, no study has evaluated the application of NNs for mono-exponential fitting of T2 relaxation times of articular cartilage using clinical framework conditions in terms of the respective imaging sequence, knee coil, and 3.0 T scanner with a clear focus to streamline and standardize post-hoc reconstructions of T2 maps.

Thus, our objective was to systematically evaluate a NN against traditional LSE fitting methods in estimating T2 relaxation times both in silico and in situ and to evaluate speed, accuracy, and robustness of each method. We hypothesized that the NN trained on synthetic data is more robust and accurate in mono-exponential T2 relaxometry than traditional LSE fitting methods, while being significantly faster and, thus, more suitable for clinical workflows.

## 2. Materials and Methods

### 2.1. Study Design

This study was conducted in two successive phases, i.e., an in silico phase and an in situ phase. First, a synthetic MRI dataset consisting of systematically varied signal intensities was generated, similar to the studies in [16,27,28], and a NN was trained on this synthetic dataset to predict T2 relaxation times, which were then compared against alternative LSE fitting methods. Second, seven human cadaver knee joints underwent T2 fitting with two distinctly different MESE sequences (Table 1). The first sequence was designed to provide high-SNR measurements that were used for reference estimations of T2 relaxation times. The second sequence was designed to provide corresponding low-SNR measurements for the subsequent evaluation of different fitting methods. The trained NN’s performance in predicting T2 relaxation times was again compared against the alternative LSE fitting methods.

Local Institutional Review Board approval (Ethical Committee, RWTH Aachen University, EK 180/16) and written informed consent by the body donors were available at study initiation. The study was performed in accordance with the relevant local guidelines and regulations.

### 2.2. In Silico Study Phase—Synthetic MRI Data

In mono-exponential T2 mapping, the magnitude of the noise-free signal intensity (*S*) at a given echo time (*TE*) is defined by $\left|S\right|=S_{0}\exp\left(-\frac{TE}{T2}\right)$, where *S*_0_ is the apparent proton density and *T*2 the voxel’s sought relaxation time. Noise is introduced by a variety of effects, mainly thermal fluctuations and electronic interference as well as dielectric and inductive losses in the patient [29]. Consequently, the signal intensity *S* is assumed to be distorted by complex white Gaussian noise $\varepsilon =\varepsilon_{real}+i\;\cdot \varepsilon_{imag}$ [30]. The real and imaginary parts of the noise follow a normal distribution with zero mean and standard deviation *σ*. The noisy signal intensity ($S_{noisy}$) is the complex addition of the noise-free signal intensity and the complex Gaussian noise as $S_{noisy}=S+\varepsilon$. In silico, the complex phase of the noise-free signal *S* was set to zero as it does not affect the magnitude of the simulated signal intensity *|S|*. Therefore, the magnitude of the noisy signal intensity was calculated as $|S_{noisy}|=\sqrt{{\left(S_{real}+\varepsilon_{real}\right)}^{2}+\varepsilon_{imag}{}^{2}}$ and will then follow the Rician distribution [30]. Note that the Rician distribution can be approximated by a Gaussian distribution for SNRs ≥3, thus justifying the widely used approach for applying LSE fitting directly to the signal magnitude data. Signal strength was obtained directly by sampling (mono-exponential) noise-free induction decays rather than a more complex Bloch simulation because the former describes the prevailing dependencies in the absence of electronic noise but without considering confounding effects such as pulse errors or diffusion, etc. [27].

**Figure A1:**
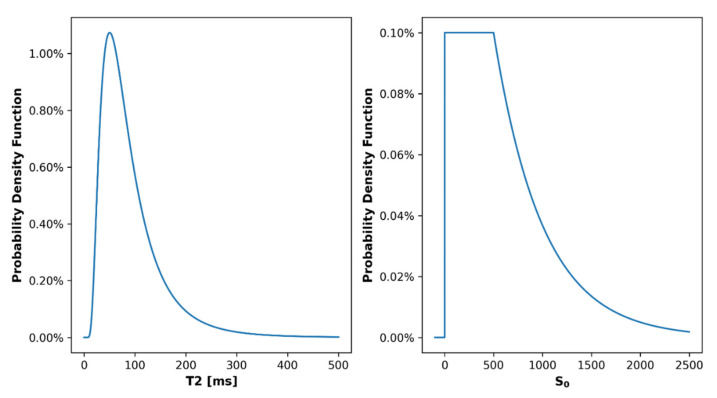
Visualization of the probability density distributions used to sample T2 relaxation times and signal intensities (*S*_0_) for the synthetic dataset. The T2 relaxation times were sampled from a log-normal distribution with $z=\frac{x-5}{70}$ and probability density function $f\left(z\right)\;\sim \frac{1}{70}\frac{1}{0.66x\;\sqrt{2\pi }}\;\exp\left(-\frac{\ln{\left(z\right)}^{2}}{2\;\cdot \;0.66^{2}}\right)$. The signal intensities followed a customized probability density function f(x), which was defined as: f(x)=0 for x<0; $f\left(x\right)=\frac{1}{1000}$ for 0≤x≤500; $f\left(x\right)=\frac{1}{1000}\exp\left(1-\frac{x}{500}\right)$ for x>500.

We systematically varied and sampled parameter distributions for ($S_{0},T2,\;TE,\;\sigma$) to generate a synthetic dataset with 67 million training samples, 8 million validation samples, and 0.5 million test samples on which the fitting procedures described below were evaluated. In this context, a sample was defined as a series of 5≤n≤15 noisy signal intensities $\left|S_{noisy}\right|\;$ as a function of *TE* ($TE_{n}=TE_{start}+n\;TE_{step}$), *S*_0_, *T*2, and *σ*. The first echo time $TE_{start}$ was sampled between 5 ms and 15 ms and the step size $TE_{step}$ between 2 ms and 15 ms. The three parameters ($TE_{start},\;n,\;TE_{step}$) were sampled from uniform distributions because no configuration was supposed to be more likely than another. In patient scans, the apparent proton density (*S*_0_) depends on many factors, including the type and configuration of the scanner, sequences, and coils used for imaging [31]. Furthermore, the apparent proton density can be scaled arbitrarily, so that previous studies defined *S*_0_ either as an arbitrary but fixed value or as a variable originating from a continuous (e.g., normal) distribution for the subsequent generation of synthetic datasets [12,32,33,34]. In reflection of these earlier studies, we defined a probability density function so that *S*_0_ values between 0 and 500 were equally likely and *S*_0_ values greater than 500 became exponentially less likely, i.e., probability P(0 ≤ *S*_0_ ≤ 500) ≈ 50%, P(0 ≤ *S*_0_ ≤ 1700) ≈ 95%, and P(0 ≤ *S*_0_ ≤ 2500) ≈ 99%. *S*_0_ was not fixed to prevent the neural network from learning or assuming a specific value for *S*_0_. For the T2 relaxation times, we assumed a log-normal distribution that is often applied to quantitative measures of living tissues [35]. Additional framework parameters were defined as follows: (i) lower threshold = 5 ms; (ii) statistical mode = 50 ms; and (iii) no fixed upper threshold but probability P(*T*2 < 210 ms) ≈ 95% and P(*T*2 < 500 ms) ≈ 99.8%. Visualizations of the underlying distributions of the T2 relaxation times and the *S*_0_ values are given in Appendix A Figure A1. Finally, to simulate different noise levels during training of the NN, it would have principally been possible (i) to vary the SNR and compute the standard deviation *σ* as *σ* = *S*_0_*/SNR* [26,36] or (ii) to vary *σ* directly. In this study, we opted for direct variation of *σ* to avoid arithmetically ill-defined constellations such as *S*_0_ = 0 or *SNR* = 0. In contrast to an earlier comparable study [37], we systematically varied the standard deviation *σ* between 0 and 300 (instead of 0 and 30), thereby accounting for the roughly 10-fold higher maximum *S*_0_ in our study.

### 2.3. In Situ Study Phase—MRI Measurements

Seven fresh-frozen human cadaver knee joints (five male and two female; mean age 81 ± 10; six left and one right) were left to thaw at room temperature for 24 h to be scanned on a 3.0 T clinical MRI scanner (Achieva, Philips, Best, The Netherlands) using an 8-channel knee coil (Sense Knee Coil 3.0T, Philips).

In this exploratory study, sample size was estimated based on the test of independence for the Mood’s median test. To this end, effect size was defined as Cohen’s *w* and estimated as 1.1. Using a statistical power of 0.8 and an alpha error of 0.05, we calculated the minimum sample size as seven.

Two different MESE T2 mapping sequences were acquired based on the sequence parameters detailed in Table 1. The sequences differed in their sensitivity encoding (SENSE) acceleration factor and their number of signal averages, which resulted in different SNRs. While the “high-SNR” T2 mapping sequence provided the signal-optimized and noise-reduced ground truth at a scan time of 26 min for one slice, the “low-SNR” T2 mapping sequence resulted in a drastically shortened scan time of 2 min for one slice at the expense of substantially increased noise. Following the acquisition of scout views, the single axial image to be acquired for each specimen was oriented parallel to the femorotibial joint line and through the center of the patella. Using the moderately T2-weighted morphologic image of *TE* = 30 ms, the outlines of the patellofemoral cartilage tissue, i.e., the retropatellar and trochlear cartilage, and of the entire knee joint’s peripheral circumference were manually delineated by GMF (medical imaging scientist with one year of experience in musculoskeletal imaging) using ITK-SNAP software [38]. SN and DT (both clinical radiologists with nine years of experience in musculoskeletal imaging) validated the segmentations.

**Table 1:** MRI acquisition parameters for the “low-SNR” and “high-SNR” multi-echo spin-echo sequences. Please note that although 10 echo times were initially sampled, only the first 7 echoes were used for the T2 fitting because of insufficient SNRs in the last echoes. The choice of echo times was guided by the Osteoarthritis Initiative study [39]. Abbreviations: MESE = Multi-echo spin-echo, SENSE = Sensitivity Encoding.

	“Low-SNR” Sequence	“High-SNR” Sequence
Sequence Type	2D MESE	
Orientation	Axial	
Repetition Time [ms]	500	
Echo Times [ms]	{10+n⋅10| n=0,1,..6}	
Field of View [mm]	140 × 140	
Acquisition Matrix	512 × 512	
Reconstruction Matrix	512 × 512	
Scan percentage [%]	100	
Flip angle [°]	90	
Number of Signal Averages [*n*]	1	4
SENSE Factor	3	1
Slices [*n*]	1	
Slice Thickness [mm]	2	
Duration [min:s]	2:15	26:34

In a voxel-wise manner, noise was estimated using variance-stabilizing transformation [40,41] and subsequent homomorphic Gaussian filtering [42]. This method estimates non-stationary noise (as in SENSE imaging) and does not require any additional information on coil sensitivity or background regions, which often hinders reliable estimation of noise [31,43]. Effective SNR values (as determined with the variance-stabilizing approach to estimate non-stationary Rician noise and averaged over all joints) were 8 ± 5 (“low-SNR” sequence) and 15 ± 9 (“high-SNR” sequence) at *TE* = 10 ms, and 5 ± 4 (low-SNR) and 10 ± 6 (high SNR) at *TE* = 70 ms. It should be noted that noise (and SNR in particular) after SENSE reconstruction is not stationary and summarizing it as a single value may not reflect the distribution and magnitude of noise.

### 2.4. Fitting Methods

Our NN was set up as a fully connected, six-layer-deep, 512-channel-wide network with Leaky Rectified Linear Unit activation functions after each layer. Only the output layer had a Rectified Linear Unit activation function since negative T2 values were not considered plausible. In total, the network comprised about 1 million trainable parameters. The signal intensities and echo times served as input. The input nodes were padded with −1 whenever less than 15 signal intensities or echo times were available as input. The batch size was set to 1024 samples and the Adam optimizer [44] with a learning rate of 10^−3^ was used. The SmoothL1 (Huber) distance between the reference and predicted parameters (*S*_0_, *T*2) served as loss function. Of note, the term “reference parameters” implies “true parameters” in the in silico setting, since the training of the NN was performed with synthetic data, where the true values of *S*_0_ and *T*2 are known a priori. This function is a combination of L1 and L2 loss and prevents exploding gradients [45]. Input samples with *S*_0_ = 0 were excluded. The NN was trained for 30 epochs, which took 36 h, and the model with the lowest loss on the validation dataset was selected for further evaluation. Python (v3.7, Python Software Foundation, Wilmington, DE, USA) and the associated libraries PyTorch and SciPy were used to implement the NN. All evaluations were performed on a dedicated graphical processing unit (Nvidia RTX 3090, 24 GB, 36TFLOPS) with a central processing unit (AMD Ryzen 9 3950X, 16 Cores, 3.5 GHz). The source code is made publicly available under https://github.com/mueller-franzes/Paper_T2Fitting (accessed on 19 January 2022).

For reference purposes, the following alternative LSE fitting methods were also implemented:

(1) Traditional LSE without any modification (LSE);
(2) Offset LSE (OLSE);
(3) Noise-Corrected LSE (NCLSE).

For the traditional LSE, OLSE, and NCLSE method, data were fitted in a voxel-wise manner to the theoretical signal intensity (*S*) by using the “curve_fit” function (SciPy). Initial values for the parameters were *S*_0_ = 250, *T*2 = 50 ms, and *c* = 0. The range (lower bound, upper bound) for *S*_0_ and *T*2 were 0, 2500 and 5 ms, 500 ms, respectively. As an optimization method, we used the Trust-Region-Reflective (SciPy ‘trf’ option) algorithm as it can solve the constrained optimization. If the least-squares minimization failed, the lower bounds were used as default.

Noise is particularly challenging for T2 quantification by traditional LSE methods as it prevents the signal from decaying to zero and causes T2 overestimation [46]. For OLSE, an additional offset parameter “*c*” was added to the exponential T2 decay to counteract the effects of noise [32,47]: $S^{*}=S_{0}\exp\left(-\frac{TE}{T2}\right)+c$.

For NCLSE, the curve was fitted to a noise-corrected exponential decay function: $S^{**}=\sqrt{0.5\pi \sigma^{2}\;}\exp\left(-\alpha \right)\left[\left(1+2\alpha \right)I_{0}\left(\alpha \right)+2\alpha \;I_{1}\left(\alpha \right)\right],$ where $\alpha ={\left(\frac{S\left(TE,T2\right)}{2\sigma }\right)}^{2}$ and $I_{n}$ is the nth modified Bessel function [12]. However, this method requires precise knowledge of noise (*σ*) in each sample. While in silico, when *σ* was known for each sample, we used voxel-wise variance-stabilizing transformation and subsequent homomorphic Gaussian filtering to estimate *σ* in the in situ knee joint measurements.

### 2.5. Computation Time

Computation times (as surrogates of computational efficiency) were determined for each fitting method, axial slice, and individual joint. Measurements were repeated 100 times and subsequently averaged. The segmentation outlines of the knee joint specimens encompassed about 130,000 voxels per knee that underwent voxel-wise quantification of T2 relaxation times based on seven TEs. The fitting methods were executed on a per-voxel basis using the central processing unit as specified above. Of note, graphical processing unit acceleration during application of the pre-trained NN was disabled.

### 2.6. Statistical Analysis

Statistical analyses were performed in Python and the associated library SciPy. Using the “low-SNR” data, T2 relaxation times were estimated for every voxel by applying the different fitting methods. For each method, deviations in T2 relaxation times were referenced to the standard LSE fitting method of the “high-SNR” data and subsequently compared between the methods. The reference standard (ground truth) was provided by the traditional LSE fitting method of the corresponding “high-SNR” images, and the voxel-wise comparisons were concatenated across all knee joint specimens. For T2 relaxometry, voxel-wise, relative quantification error ($RQE=\frac{T2_{pred}-T2_{ref}}{T2_{ref}}\cdot 100$) was calculated and visualized as box plots. For RQEs, the interquartile ranges (IQRs) were determined as a metric of variability in T2 quantification. Positive median RQEs indicate overestimation of the reference T2 relaxation times, while negative median RQEs indicate underestimation. Additionally, absolute-valued relative quantification errors ($ARQE=\frac{\left|T2_{pred}-T2_{ref}\right|}{T2_{ref}}\cdot 100$) were calculated to prevent cancelation of positive and negative relative errors. Based on the test for normality by D’Agostino and Person, we had to reject the hypothesis of normally distributed ARQES and RQEs. Hence, median (instead of mean) ARQEs were computed to minimize the influence of outliers. Median ARQEs were interpreted as a metric of accuracy in T2 quantification. Mood’s median test was performed to compare the median ARQEs of the different fitting methods. This test was chosen because more powerful tests such as the Mann–Whitney U-test may fail when comparing medians instead of means [48]. Mean computation times were compared between the NN and the LSE methods using the one-sided Wilcoxon signed-rank test. To prevent alpha-error inflation and, thus, inflation of the false positive rate, the significance threshold was lowered to *α* = 0.05/3 = 0.0166 [49] because post-hoc comparisons were performed only between the NN and the three fitting methods, i.e., NN vs LSE, NN vs. OLSE, and NN vs. NCLSE.

## 3. Results

### 3.1. In Silico Fitting Results

**Figure 1:**
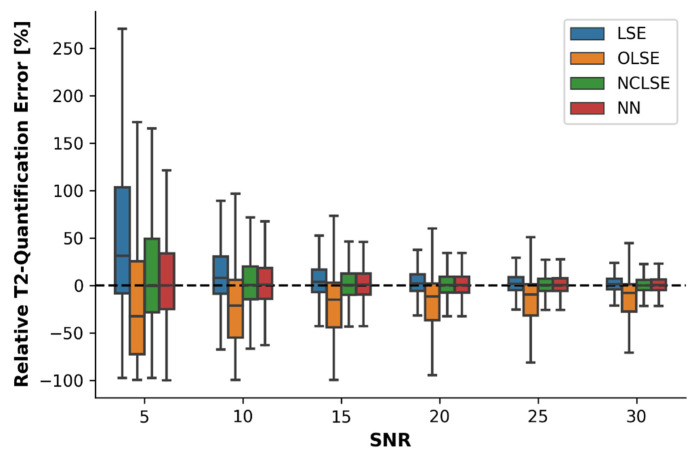
Relative quantification errors (RQEs) in the quantification of T2 relaxation times [%] as a function of the fitting method, i.e., the traditional Least-Squares Estimation (LSE) without any further modification, Offset LSE (OLSE), Noise-Corrected LSE (NCLSE), and the neural network (NN), and as a function of the signal-to-noise ratio (SNR). In silico modeling was done on a synthetic dataset consisting of 67 million training samples. Boxes represent the interquartile ranges (IQRs, defined as the difference between 25th and 75th percentiles) and horizontal lines represent the medians. Whiskers indicate the most extreme data points that are within the range of 1.5 × IQR from the edge of the box.

In silico modeling indicated that RQEs decreased as a function of increasing SNR, irrespective of the fitting method (Figure 1). Especially in low-SNR environments (i.e., SNR ≤ 10), LSE overestimated the T2 relaxation times as indicated by positive median RQEs (e.g., median RQE = 31% at SNR = 5). The opposite was true for OLSE, which underestimated the T2 relaxation times as indicated by negative median RQEs (e.g., median RQE = −33% for SNR = 5). In contrast, the median RQEs of NCLSE and NN were centered around 0% for all SNRs, indicating bias-free estimations. In high-SNR environments, i.e., SNRs ≥ 30, the median RQEs of all fitting methods were between 0% and 1%, except for OLSE (median REQ = −8%).

These findings were confirmed by the ARQE values (Table 2). While all fitting methods were characterized by large ARQEs at low SNR, ARQEs gradually decreased with increasing SNR. The NN was characterized by the lowest ARQE, indicating highest accuracy, for all sampled SNRs ≤ 20. Especially at low SNRs, i.e., SNR ≤ 10, the NN demonstrated significantly lower median ARQEs compared to the LSE, OLSE, and NCLSE methods (Mood’s Test, *p* < 0.001). With higher SNRs (≥20), the median ARQEs for LSE, NCLSE, and NN were largely similar, with ranges of 8–9% (SNR = 20) and 5–6% (SNR = 30), respectively. Only the ARQEs for OLSE were twice as high.

### 3.2. In Situ Fitting Results

**Figure 2:**
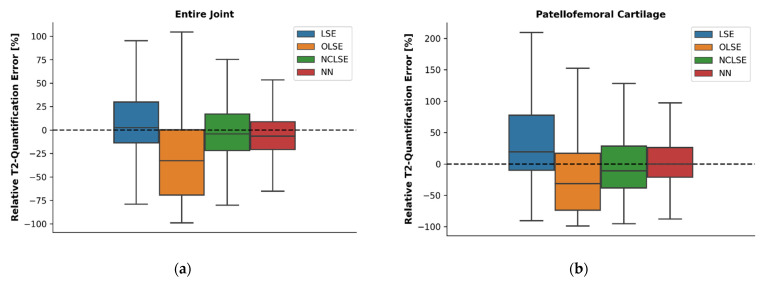
Relative T2 quantification errors (RQEs) [%] as a function of fitting method in the entire joint (**a**) and the patellofemoral cartilage (**b**) across all seven knee joint specimens in the “low-SNR” data, computed with respect to the reference T2 relaxation times. The reference T2 relaxation times were estimated using the LSE fitting method and the corresponding “high-SNR” image. For an explanation of the boxes, lines, and whiskers please refer to Figure 1.

In situ fitting results of the entire knee joint and the patellofemoral cartilage were largely in line with the in silico fitting results outlined above. Again, the worst performance (in terms of RQE) was noted for the OLSE, which underestimated the T2 relaxation times by −33% (entire knee joint) and −31% (patellofemoral cartilage), respectively (Figure 2). The LSE method overestimated the T2 relaxation times by +2% and +19%, respectively. The NN and the NCLSE provided the best estimates of the T2 relaxation times (in terms of lowest RQEs) in both regions. While medians were similar, the NN provided less variable estimates, as indicated by lower IQRs.

Correspondingly, median ARQEs and associated IQRs were smallest for the NN in the entire joint and the patellofemoral cartilage (Table 3). These differences were significant when comparing the NN to the LSE (Mood’s Test, *p* < 0.001), OLSE (*p* < 0.001), and NCLSE (*p* < 0.001).

**Figure 3:**
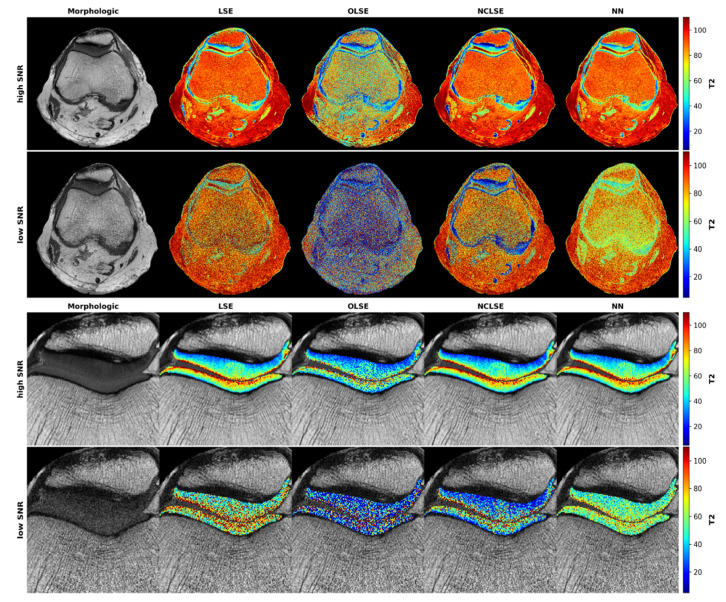
Representative T2 maps as a function of signal-to-noise ratio (SNR) and underlying fitting method. Visualization of the axial plane acquired at high SNR (first row) and at low SNR (second row) of the entire joint that was cropped and zoomed to the patellofemoral compartment (third and fourth rows for high- and low-SNR images) in this representative knee joint. The first column shows the T2-weighted morphologic images (TE = 30 ms). The second to fifth columns visualize the T2 maps following fitting based on the Least-Squares Estimation (LSE, second column), Offset LSE (OLSE, third column), Noise-Corrected LSE (NCLSE, fourth column), and the Neural Network (NN, fifth column). T2 relaxation times [ms] are color-coded as indicated by the scale bars on the right (range: 0–110 ms).

**Figure A2:**
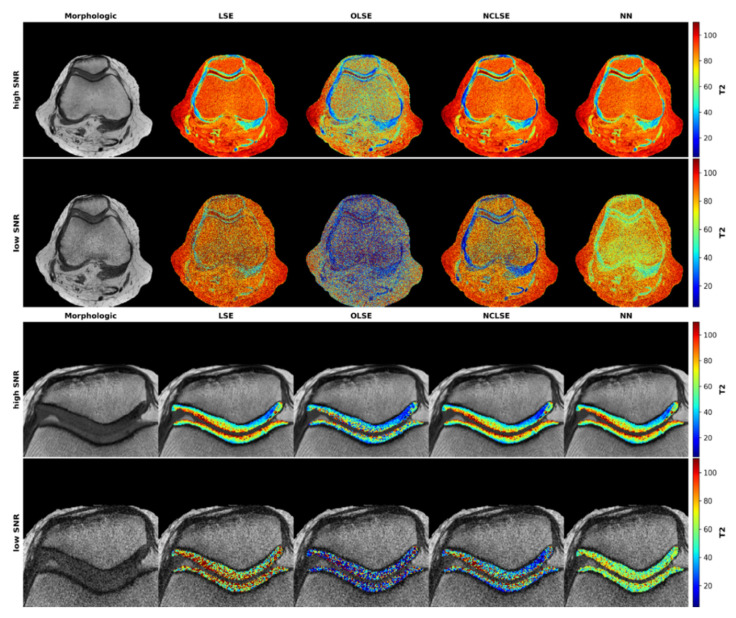
Representative T2 maps as a function of signal-to-noise ratio (SNR) and underlying fitting method. Visualization of the axial plane acquired at high SNR (first row) and at low SNR (second row) of the entire joint that was cropped and zoomed to the patellofemoral compartment (third and fourth rows for high- and low-SNR images) in this representative knee joint. The first column shows the T2-weighted morphologic images (*TE* = 30 ms). The second to fifth columns visualize the T2 maps following fitting based on the Least-Squares Estimation (LSE, second column), Offset LSE (OLSE, third column), Noise-Corrected LSE (NCLSE, fourth column), and the Neural Network (NN, fifth column). T2 relaxation times [ms] are color-coded as indicated by the scale bars on the right (range: 0–110 ms).

Qualitative evaluation revealed that in cartilage, the characteristic T2 stratification as a function of cartilage depth was visible in all high-SNR T2 maps, regardless of the underlying fitting procedure, even though OLSE-fitted T2 maps tended to display larger variability in pixel distribution and intensity (Figure 3). In contrast, low-SNR T2 maps displayed substantial blurring, which rendered depth-wise intra-tissue stratification and areas of focal degeneration barely discernible. For the patellofemoral cartilage, closest correspondence with the reference high-SNR T2 maps (which were fit with the traditional LSE method) was found for the NCLSE and the NN. These results were confirmed in other knee joints as well (Appendix A Figure A2).

### 3.3. Computation Time

Mean computation times of the fitting methods were significantly different (Table 4). It took the NN 4 s to compute the single axial T2 map, which was significantly faster than the 28–43 s of the LSE methods (Wilcoxon test, *p* < 0.001). On average, the NN was 600%, 975%, and 900% faster than the LSE, OLSE, and NCLSE, respectively.

## 4. Discussion

The most important finding of this study is that an NN can estimate T2 relaxation times significantly more accurately and quickly in low SNR environments than traditional LSE methods. Most importantly, the NN derives its estimates of T2 relaxation times from a standard MESE T2 mapping sequence and does not necessitate the acquisition of dedicated MR sequences or other modifications to the imaging protocol. This confirms our hypothesis, that a NN is more robust and accurate in mono-exponential T2 relaxometry than traditional LSE fitting methods while being significantly faster and, thus, more suitable for clinical workflows. Consequently, NN-based approaches may become a valid tool to improve image post-processing routines in quantitative cartilage imaging and beyond. For this purpose, the NN and the LSE methods were analyzed in silico (i.e., on a synthetic dataset) and in situ (i.e., in human knee joint specimens).

It is well known that the traditional LSE method is prone to outliers and its fit quality is substantially impaired in low SNRs [18,19], which was confirmed in our study. For all simulated SNRs, the traditional LSE method performed worse (up to 15% higher ARQEs) than the NN. The results also show that the traditional LSE method overestimates T2 relaxation times by up to 31%, while the NN provides the least biased in silico estimates. In our simulations, this behavior was particularly evident for comparatively low SNRs, i.e., SNRs ≤ 10. As Rician noise will cause bias once the actual signal has decayed, this observation aligns well with other studies [32,46].

Adding an offset as a third parameter to the mono-exponential decay (which we defined as the “OLSE method”) was intended to prevent this overestimation. However, our in silico and in situ results showed that the OLSE method was characterized by underestimation of the reference T2 relaxation times. Overall, the T2 quantification error was higher compared with the traditional LSE method in this study. Even though the finding of increased T2 quantification errors is in line with earlier studies [33], other studies found the opposite [32]. A possible explanation for these contradictory results is that the additional offset parameter as provided by the OLSE method becomes particularly useful when T2 relaxation times are small compared with the covered range of echo times and when noise levels are high, but may cause underestimation when T2 relaxation times are long (12, 39). Thus, the benefit of introducing an additional offset during fitting depends on the exact framework conditions. These observations are in line with an earlier study by Raya et al. [12], who noted that the additional offset parameter improved the quantification accuracy in healthy cartilage in voxels with short T2 relaxation times, but led to severe underestimations in voxels with long T2 relaxation times. These aspects are noteworthy given the fact that the OLSE method is widely used [17,33].

Another modification of the traditional LSE method, i.e., the introduction of additional noise correction to the exponential decay function (which we defined as the “NCLSE method”), resulted in improved accuracy and lower variability, both in silico and in situ, which is in line with earlier studies [12,27,50]. It should be underlined that the noise level needs to be provided for the NCLSE method, which was realized using the variance-stabilizing approach to estimate non-stationary Rician noise as published by Pieciak et al. [41]. This method has some major advantages over alternative SNR estimation methods (such as providing local SNR estimates and stable results over a wide range of SNR values while not requiring coil sensitivity maps or knowledge on the reconstruction algorithm). Nevertheless, an additional noise estimation that may add uncertainty when performed in situ [51] and increases computation time is not necessary for the NN, which is advantageous.

In situ, the NN’s median ARQE was significantly lower than the ARQEs of the LSE, OLSE, and NCLSE methods. The higher accuracy and lower variability afforded by the NN was particularly evident in low SNRs, which indicates its diagnostic potential, as clinical MRI studies are usually characterized by suboptimal SNRs secondary to trade-offs between imaging speed and image quality. In situ, the traditional LSE performed better than the OLSE but worse than the NCLSE. Overall, these findings were consistent with the in silico results outlined above. We would like to emphasize that our measured in situ data did not cover all possible combinations of T2 relaxation times, TEs, and SNRs. Furthermore, the in situ results confirmed that the LSE and OLSE method tended to over- and underestimate the actual T2 relaxation times at low SNR, respectively, while the NCLSE and NN provided more robust and less biased estimates in comparison to the reference T2 relaxation times obtained at high SNR with the traditional LSE method. In addition to accuracy, variability, and robustness, the trained NN was also characterized by significantly lower post-processing time demand as it was 600% faster than the fastest LSE method. Of course, computation times depend on numerous framework conditions such as hardware components and the implementation of the algorithms. Regardless of these considerations, once the NN is trained, execution does not require any time-consuming, incremental optimization.

The T2 maps of the high-SNR sequences demonstrated the typical stratification of the T2 relaxation times that ranged between 20 ms and 60 ms with lower values towards the cartilage-bone interface and higher values towards the cartilage-fluid interface, regardless of the underlying fitting procedure. However, substantially higher T2 relaxation times were observed at the superficial cartilage layer. These are most likely due to structural disintegration and degeneration or partial volume effects. In lack of histologic (or other) references, the exact correlate of the extended T2 value ranges remains unclear. However, because the same segmentation outline was used for all fitting techniques, inter-method comparisons are still permissible and valid.

Beyond T2 mapping, NNs may be used to predict virtually any signal decay in the post-processing of MRI signals and could be applied to T1ρ, T2*, glycosaminoglycan chemical exchange saturation transfer imaging, and sodium imaging in the context of cartilage imaging [52,53,54]. In light of the research community’s increasing collaborative efforts to identify imaging biomarkers for cartilage degeneration, such as the Osteoarthritis Initiative [39], the Multicenter Osteoarthritis Study [55], and others, the need for more reliable and efficient post-processing to decrease inter-individual and inter-site variability becomes ever more urgent [56]. Our findings suggest that pre-trained NNs may be interesting tools for improved standardization of image post-processing once they have been refined for large-scale clinical trials.

Our study has several major limitations. First, the evaluation was carried out on cadaver knee joint specimens only. We intentionally performed the measurements in situ (and not in vivo) to securely eliminate any (phase-encoded) motion artifacts during the lengthy high-SNR measurements. Future studies need to confirm the principal in vivo applicability of our method, where arterial pulsations or physical movement certainly increase the number of outliers and affect the fitting accuracy and variability.

Second, our evaluation was limited to seven knee joint specimens, which may have satisfied statistical considerations on minimum sample sizes but provided only limited in situ data. Our synthetic dataset was designed to incorporate different choices of echo times, yet was evaluated on one specific T2 mapping sequence and one MRI scanner only. Further evaluation is needed to see whether these methods can be applied across the large variety of available MRI sequences, scanners, and coils. On top of that, future work should evaluate the precision of the different algorithms to prove if the NN provides superior performance over the LSE methods [57]. This includes, but is not limited to, testing repeatability.

Third, the comparative evaluation of quantification errors in situ required referencing the high-SNR measurement (which was fitted using the traditional LSE method) as the ground truth. It should be noted that this reference may be prone to residual noise, which may affect the estimated T2 relaxation times used for reference purposes. While the exact amount of over- or underestimation in T2 quantification, thus, remains unclear, the in situ results corroborate the in silico findings, as detailed above. It is worth mentioning that both synthetic data and phantom knees can enable comparison to known, ground truth values [57]. Admittedly, experiments using a standardized quantitative knee phantom would have been desirable for further validation but were not performed in this study because a suitable knee phantom was not available.

Fourth, the MESE sequences are insufficient for assessing the short and very short T2 components present adjacent to the calcified cartilage and subchondral lamella. Ultrashort echo-time sequences are diagnostically beneficial for the assessment of very short T2 relaxation times [58], yet their comprehensive assessment is beyond the scope of this study. Once ultrashort echo-time sequences are used in the future, the NN ought to be re-trained in silico with a focus on T2 relaxation times ≤ 10 ms. Furthermore, MESE sequences are susceptible to confounding influences such as simulated echoes, the slice profile or flip angles deviating from the refocusing pulse [13,14,15]. Nevertheless, MESE sequences are traditionally combined with standard LSE fitting approaches and provided on most clinical scanners, and, hence, relevant for clinical practice and research [39,59].

Fifth, the NN was not compared to alternative deep learning-based methods for T2 quantification, e.g., [24,25,26,60]. Instead of aligning T2 maps with deep learning to provide tools for cartilage segmentation or data augmentation for subsequent T2 quantification, our neural network was pre-trained on synthetic datasets and is, thus, more independent of any particular image acquisition and post-processing technique. Consequently, its performance was evaluated against the traditional LSE method (and its refinements) as the current standard approach in a proof-of-concept study. Comprehensive comparison with other deep learning-based methods remains to be addressed in future studies. Even though, principally, the NN’s excellent fitting performance has validated the synthetic dataset used for its training in silico, more advanced signal simulation methods, such as Bloch simulations, that consider the effects of diffusion or pulse errors, could further improve its performance. In our study, a fully connected NN was used to fit the T2 relaxation times in a voxel-wise manner. It is possible that a convolutional NN may perform even better when set up to provide T2 estimates in a patch-based manner. Neighboring pixels contain valuable information on signal and noise that could be used for more accurate estimates in future studies. Training, however, would require extended amounts of synthetic data for realistic spatial noise distributions and similar T2 relaxation times.

## 5. Conclusions

We have trained a neural network to provide fast, accurate, and robust quantification of T2 relaxation times, in particular in low SNR environments.

## Figures and Tables

**Table 2:** Median absolute-valued relative quantification errors (ARQE) in the quantification of T2 relaxation times [%] as a function of fitting method and SNR. Data are given as medians [2.5th percentile, 97.5th percentile]. Please refer to Figure 1 for an explanation of the abbreviations.

	SNR = 5	SNR = 10	SNR = 20	SNR = 30
LSE	43 [2, 650]	19 [1, 199]	9 [0, 63]	6 [0, 39]
OLSE	61 [3, 439]	33 [1, 115]	17 [1, 94]	11 [0, 90]
NCLSE	34 [2, 509]	17 [1, 175]	8 [0, 61]	5 [0, 39]
NN	28 [1, 160]	16 [1, 83]	8 [0, 47]	6 [0, 34]

**Table 3:** Median absolute-valued relative quantification errors in the quantification of T2 relaxation times [%] as a function of fitting method. Data are given as medians [2.5th percentile, 97.5th percentile]. Please refer to Figure 1 for an explanation of the abbreviations.

	Entire Joint	Patellofemoral Cartilage
LSE	19 [1, 500]	33 [1, 651]
OLSE	47 [2, 236]	58 [2, 398]
NCLSE	20 [1, 381]	35 [1, 513]
NN	16 [1, 79]	23 [1, 120]

**Table 4:** Mean computation times [s] to compute a single T2 map of the entire joint. Standard deviation was below 1 s for all fitting methods (calculated over 100 repetitions of the fitting process). Abbreviations are defined in Table 1.

	LSE	OLSE	NCLSE	NN
Computation Time [s]	28	43	40	4

## Data Availability

The source code of this manuscript has been made publicly available on GitHub: https://github.com/mueller-franzes/Paper_T2Fitting (accessed on 19 January 2022).
